# Detection of HIV-1 RNA/DNA and CD4 mRNA in feces and urine from chronic HIV-1 infected subjects with and without anti-retroviral therapy

**DOI:** 10.1186/1742-6405-6-20

**Published:** 2009-10-02

**Authors:** Ayan K Chakrabarti, Lori Caruso, Ming Ding, Chengli Shen, William Buchanan, Phalguni Gupta, Charles R Rinaldo, Yue Chen

**Affiliations:** 1 Department of Infectious Diseases and Microbiology, Graduate School of Public Health, University of Pittsburgh, Pittsburgh, Pennsylvania 15261, USA

## Abstract

HIV-1 infects gut associated lymphoid tissues (GALT) very early after transmission by multiple routes. The infected GALT consequently serves as the major reservoir for HIV-1 infection and could constantly shed HIV-1 and CD4^+ ^T cells into the intestinal lumen. To examine this hypothesis, we monitored HIV-1 RNA/DNA and CD4 mRNA in fecal samples of chronically infected subjects with and without antiretroviral therapy (ART). We compared this to levels of HIV-1 RNA/DNA in urine and blood from the same subjects. Our results show that HIV-1 DNA, RNA and CD4 mRNA were detected in 8%, 19% and 31% respectively, of feces samples from infected subjects with detectable plasma viral load, and were not detected in any of subjects on ART with undetectable plasma viral load. In urine samples, HIV-1 DNA was detected in 24% of infected subjects with detectable plasma viral load and 23% of subjects on ART with undetectable plasma viral load. Phylogenetic analysis of the envelope sequences of HIV-1 revealed distinct virus populations in concurrently collected serum, feces and urine samples from one subject. In addition, our study demonstrated for the first time the presence of CD4 mRNA in fecal specimens of HIV-1 infected subjects, which could be used to assess GALT pathogenesis in HIV-1 infection.

## Introduction

Gut-Associated Lymphoid Tissues (GALT) are very important in HIV pathogenesis. GALT is the largest single immunologic organ in the body, containing a large amount of lymphocytes. Contrast to the blood and other organized lymphoid tissues, which contain abundance of naive resting T cells, a majority of the CD4^+ ^T cells that reside in GALT are CCR5 positive, activated memory CD4 T cells which are the preferred target cells for HIV/SIV infection [1-3]. HIV infects GALT at a very early stage of infection regardless of the route of infection and active HIV/SIV replication in GALT is present throughout the entire course of infection, which leads to GALT acting as a major viral reservoir and results in mucosal barrier dysfunction and bacterial translocation that contributes to generalized systemic immune activation and disease progression[2-6].

CD4^+ ^T cells in the gut are rapidly infected and depleted soon after infection[3,7] and CD4^+ ^T cell repopulation of the gut is prevented throughout infection[4]. We hypothesize that during HIV-1 infection, HIV-1 free virus and infected/uninfected CD4^+ ^T cells constantly shed from GALT into the intestinal lumen and are discharged with feces. Therefore, the amount of HIV-1 and CD4^+ ^T cells contained in the feces could reveal the degree of pathogenesis in GALT. Detection of HIV-1 has been reported in fecal specimens from drug naïve HIV-1 infected individuals in the acute phase of infection [8-10]. There is no information on HIV-1 detection in the feces of chronically infected subjects, especially in subjects undergoing antiretroviral drug therapy (ART). Monitoring the dynamic change of CD4^+ ^T cells in GALT of infected individuals is very important to evaluate disease progression. However, due to the anatomic location of GALT, invasive and expensive biopsy is the only current method to monitor CD4^+ ^T cell loss in GALT. In contrast, feces could be an easily accessible, non-invasive and inexpensive specimen to assess CD4^+ ^T cell depletion of GALT, since CD4^+ ^T cells could shed into the intestinal lumen and be discharged in feces.

HIV-1 from seropositive individuals has been detected from various body fluids including blood, semen, tears, saliva, cerebrospinal fluid, breast milk and cervical secretions[11]. A broad spectrum of renal diseases has been reported in HIV-1 infected AIDS subjects [12-14], yet there is little information on the presence of HIV-1 in urine. The presence of anti-HIV-1 antibodies has been reported in urine by ELISA and Western blot [15,16] and HIV-1 DNA has been detected in urine pellets from HIV-1 infected individuals [17,18]. However, it is not clear whether urine from chronically infected persons with/without ART contains HIV-1 DNA/RNA, and how virus in urine is related to the viral load in serum.

In this study, we detected HIV-1 RNA/DNA in fecal and urine specimens from chronically HIV-1 infected subjects with or without ART. In addition, we examined the presence of human CD4 mRNA in fecal specimens to assess CD4^+ ^T cell loss in GALT.

## Materials and methods

### Study Participants

The uninfected and HIV-1 infected subjects in this study were enrolled in the Multicenter AIDS Cohort Study (MACS) at Pittsburgh, PA. The MACS is an ongoing prospective natural history study of HIV-1 infection in homosexual and bisexual men enrolled at Baltimore, Chicago, Pittsburgh and Los Angeles. The study was approved by the University of Pittsburgh Institutional Review Board (IRB). Fecal specimens that were used in this study were collected in 2008. Thirty-nine samples were collected from the subjects in four different groups: Group A: HIV-1 negative; Group B: HIV-1 positive but not on ART; Group C: HIV-1 positive on ART with non-detectable viral load in blood; Group D: HIV-1 positive on ART with detectable viral load in blood (Table 1).

**Table 1:** Clinical information of 2008 MACS study participants

	**ID**	**Sample Date**	**Viral Load (copies/ml)**	**CD4/mm3**
**Group A = HIV-1 Negative N = 10**	XX110	8/27/2008	N/A	642
	
	XX712	8/16/2008	N/A	1317
	
	XX163	8/19/2008	N/A	899
	
	XX983	8/19/2008	N/A	1153
	
	XX271	9/5/2008	N/A	828
	
	XX003	9/16/2008	N/A	547
	
	XX744	9/25/2008	N/A	1520
	
	XX186	9/24/2008	N/A	839
	
	XX148	9/17/2008	N/A	724
	
	XX021	8/19/2008	N/A	845

**Group B = HIV-1 Positive/No antiretroviral treatment (ART) N = 11**	XX280	8/26/2008	1003	923
	
	XX495	8/16/2008	35471	238
	
	XX326	8/23/2008	20149	392
	
	XX286	9/17/2008	2974	320
	
	XX119	8/26/2008	18231	301
	
	XX200	8/26/2008	58200	149
	
	XX305	9/4/2008	582	388
	
	XX053	9/26/2008	78636	406
	
	XX109	9/25/2008	428	358
	
	XX013	9/12/2008	20441	457
	
	XX634	9/18/2008	6779	352

**Group C = HIV -1 Positive/ART/Non-detectable viral load N = 13**	XX484	8/19/2008	<50	440
	
	XX008	8/20/2008	<50	890
	
	XX523	9/5/2008	<50	696
	
	XX245	9/23/2008	<50	566
	
	XX163	9/10/2008	<50	549
	
	XX690	9/9/2008	<50	385
	
	XX005	8/28/2008	<50	777
	
	XX144	8/28/2008	<50	583
	
	XX154	8/20/2008	<50	922
	
	XX327	9/30/2008	<50	426
	
	XX350	9/3/2008	<50	466
	
	XX263	9/17/2008	<50	697
	
	XX265	9/25/2008	<50	478

**Group D = HIV-1 Positive/ART detectable viral load N = 5**	XX127	9/3/2008	694	161
	
	XX229	8/28/2008	186	540
	
	XX371	9/10/2008	33751	152
	
	XX099	9/23/2008	842	153
	
	XX274	9/9/2008	16842	279

### Collection and storage of biological specimens

Fecal samples were collected in special stool collection tubes (Sarstedt) and were stored in RNAlater solution (Ambion) or Cell-Lysis buffer in -80C freezer within 6 hours of collection. Urine samples were processed within 6 hours of collection. Blood samples were collected from all study participants at the same time as feces and urine. Plasma, serum and PBMC were isolated from these blood samples and used for CD4^+ ^T cell counts and viral load measurement.

### HIV-1 infected cell line and HIV-1 positive plasma

The 8E5 cell line used in this study is derived from HIV-1 infected CD4^+ ^CEM cells, and carries a single, integrated and RT-defective HIV-1 genome[19]. The HIV-1 positive plasma with viral load of 170,000 copies/ml was obtained from a HIV-1 (subtype B) infected Brazilian blood donor. 8E5 cell line and HIV-1 positive plasma were added to feces from HIV-1 negative persons before nucleic acid isolation to test the detection limit of HIV-1 DNA/RNA by PCR.

### Extraction of RNA/DNA from feces samples

Two hundred milligrams of feces with or without 8E5 cells was used to isolate RNA/DNA with a nucleic acid isolation kit from Biomerieux following the manufacturer's instructions. Briefly, specimens stored in 2 ml Cell-Lysis buffer were thawed and mixed completely by vortexing. Fifty microliters of silica bead suspension was added to the fecal sample and the sample mixture was incubated at room temperature for 10 min. with periodic vortexing and centrifuged for 3 minutes at 1500 g. The supernatant was removed and the pellet was washed five times: 2 times with wash buffer, 2 times with 70% grade ethanol and 1 time with analytical grade acetone. The silica-nucleic acid complexes were dried on a heat block at 56°C for 10 minutes and nucleic acids were eluted using 100 ul of elution buffer. Eluted nucleic acids were immediately stored at -70°C for further use.

### Extraction of RNA/DNA from urine samples

26-83 ml of urine were collected from the study participants and processed within 6 hours. The urine samples were centrifuged at 1500 g for 10 minutes at 4°C. The urine pellets were saved in -80°C for DNA isolation. The urine supernatant was concentrated by a Centricon plus-70 filter with molecular weight cutoff 100 kDa (Millipore) according to manufacturer's instruction. Briefly, urine supernatant was centrifuged in a pre-wet Centricon at 500 g for 1.5 hrs and the concentrated supernatant was collected by inverted spinning and further concentrated by ultracentrifugation at 22,000 rpm for one hour at 4°C. Most of the supernatant was removed and 50 ul of remaining supernatant and pellet was saved at -80°C for further RNA isolation.

RNA was purified from the remaining supernatant and pellet after ultracentrifugation using RNA-Bee RNA Isolation kit (Tel-Test) according to manufacturer's instruction. Briefly, 1 ml of RNA-Bee solution and 200 ul of chloroform were added to the sample and shaken vigorously for 30 seconds at room temperature. Then, the sample was incubated in 4°C for 5 minutes and centrifuged at 12,000 g for 15 minutes at 4°C. After centrifugation, the aqueous phase containing RNA was carefully recovered. The RNA was precipitated using isopropanol, washed with 75% ethanol, air dried and stored in nuclease free water at -80°C for future RT-PCR work.

DNA was purified from the urine pellet using PUREGENE DNA Purification Kit (Gentra Systems) according to manufacturer's instruction. Briefly, urine pellet from initial centrifugation was resuspended in 900 ul of Cell Lysis Solution and incubated at 65°C for 30 min. After incubation, 5 ul of RNase A Solution was added to the mixture and incubated at 37°C for 30 min. Then, 300 ul of Protein Precipitation Solution was added to the mixture and vortexed followed by centrifugation at 2000 g for 10 minutes. DNA was precipitated from the supernatant by isopropanol, washed by 70% ethanol and air dried. The DNA was re-hydrated in nuclease free water and stored at -20°C for subsequent PCR work.

### Nested PCR and RT-PCR

Nested PCR and RT-PCR were performed on isolated RNA/DNA samples from feces and urine to detect HIV-1 RNA/DNA and CD4 mRNA. Specific primers listed in Table 2 were used for optimal detection of HIV-1 subtype B *env *and *gag *regions, human beta-globin DNA, human beta-actin mRNA and CD4 mRNA.

**Table 2:** The primers used for nested PCR/RT nested PCR amplification

**Name**	**Sequences (5'→3')**	**Description**
ED12	AGT GCT TCC TGC TGC TCC CAA GAA CCC AAG	RT primer for HIV env gp120

ED31	CCT CAG CCA TTA CAC AGG CCT GTC CAA AG	1^st ^round PCR forward primer for HIV env gp120

BH2	CCT TGG TGG GTG CTA CTC CTA ATG GTT CA	1^st ^round PCR reverse primer for HIV env gp120

DR7	TCA ACT CAA CTG CTG TTA AAT GGC AGT CTA GC	2^nd ^round PCR forward primer for HIV env gp120

DR8	CAC TTC TCC AAT TGT CCC TCA TAT CTC CTC C	2^nd ^round PCR reverse primer for HIV env gp120

Hu-CD4 RT primer	ATG TCT TCT GAA ACC GGT GAG GAC ACT G	RT primer for human CD4 mRNA

Hu-CD4 outside F	CCA AGT CTT GGA TCA CCT TTG ACC TGA AG	1^st ^round PCR forward primer for Human CD4 cDNA

Hu-CD4 outside R	AGA AGA AGA TGC CTA GCC CAA TGA AAA GC	1^st ^round PCR reverse primer for Human CD4 cDNA

Hu-CD4 Inside F	CTC CCG CTC CAC CTC ACC CTG	2^nd ^round PCR forward primer for Human CD4 cDNA

Hu-CD4 Inside R	CAT GTG GGC AGA ACC TTG ATG TTG G	2^nd ^round PCR reverse primer for Human CD4 cDNA

B-globin outside F	CTG CTG GTG GTC TAC CCT TGG AC	1^st ^round PCR primer for Human Beta globin DNA

B-globin outside R	CTC AAG TTC TCA GGA TCC A	1^st ^round PCR primer for Human Beta globin DNA

B-globin inside F	GGT TCT TTG AGT CCT TTG GGG ATC	2^nd ^round PCR forward primer for Human Beta globin DNA

B-globin inside R	GTC ACA GTG CAG CTC ACT CAG TGT G	2^nd ^round PCR reverse primer for Human Beta globin DNA

B-actin outside F	GCA CCA CAC CTT CTA CAA TG	1^st ^round PCR primer for Human Beta actin cDNA

B-actin outside R	TGC TTG CTG ATC CAC ATC TG	1^st ^round PCR primer for Human Beta actin cDNA

B-actin inside F	TAC CAC TGG CAT CGT GAT GGA CTC	2^nd ^round PCR primer for Human Beta actin cDNA

B-actin inside R	CGC TCA TTG CCA ATG GTG ATG AC	2^nd ^round PCR primer for Human Beta actin cDNA

Gag outside F	GGC CAT ATC ACC TAG AAC TTT AAA TGC ATG G	1^st ^round PCR primer for HIV Gag

Gag outside R	CCT ACT GGG ATA GGT GGA TTA TTT GTC ATC CA	1^st ^round PCR primer for HIV Gag

Gag inside F	GGC ACA TCA AGC AGC CAT GCA AAT G	2^nd ^round PCR primer for HIV Gag

Gag inside R	TAG TTC CTG CTA TGT CAC TTC CCC TTG G	2^nd ^round PCR primer for HIV Gag

First a cDNA strand was generated using Superscript II RT (Invitrogen, Carlsbad, CA). A 10 ul reaction consisting of 10 ug of RNA, 2 uM of primer, 10 mM dNTP mix, and H2O was incubated at 70°C for 10 minutes. Following incubation, 5× RT buffer, 0.1 M DTT, RNA guard (RNase) (40 U/ul), and Superscript II RT (200 U/ul) were added respectively. The reaction was incubated in a H_2_O bath at 42°C for 50 minutes followed by a second incubation in dry bath at 70°C for 10 minutes. Amplification of specific target sequences in the cDNA was performed using 10 μl cDNA, forward and reverse primer pairs, dNTPs, Taq polymerase buffer and Taq-polymerase in thermocycler (Applied Biosystems) with cycling conditions of 94°C, 10 min followed by 35 cycles of 94°C, 1 min, 55°C, 1 min, 72°C, 1 min. Presence of the amplicon was analyzed on a 1%-2.5% agarose gel in 1× TAE buffer.

### Detection of fecal occult blood from feces samples

The fecal samples were tested for presence of occult blood by a Hemoccult II SENSA kit (Beckman-Coulter). Trace amount of the fecal sample was smeared onto an absorbent paper that has been treated with a chemical guaiac. Hydrogen peroxide was dropped onto the fecal smear. If trace amounts of blood were present, a blue color developed. A total of 39 fecal specimens from the study participants (Table 1) were tested. In addition, a normal donor fecal sample was included as a negative control and normal donor fecal sample mixed with blood as a positive control.

### Cloning and sequencing of PCR products

The PCR product was purified from an agarose gel and ligated into TOPO vectors (Invitrogen, Carlsbad, CA) according to manufacturer's instruction. Cloned plasmid DNA was purified from transformed *E. Coli *using Wizard^® ^Plus Minipreps DNA Purification System (Promega, Madison, WI) and digested with EcoRI restriction enzyme to confirm the insertion. The plasmid DNAs with the insertions were then sequenced using M13 forward or M13 reverse primers. Sequences were assembled and error checked using the Vector NTI 9.0 software (Invitrogen) and aligned with reference sequences from GenBank by the ClustalW multiple sequence alignment programs from Mega 4.0.

## Results

### Evaluation of the sensitivity of the PCR detection of HIV-1 DNA/RNA in human feces

To test the sensitivity of amplifying HIV-1 DNA, 200 mg of normal donor fecal sample was mixed with different concentrations of HIV-1 positive 8E5 cells. Nucleic acid was isolated using NUCLISENS (BIOMERIEUX) nucleic acid isolation kit. For every DNA sample, the human beta-globin gene was PCR amplified to ensure that the isolated DNA was amplifiable and contained the comparable amount of human DNA. Subsequently, a nested PCR reaction was performed to detect HIV-1 DNA from the isolated DNA using HIV-1 env specific primers. HIV-1 DNA was detected from the DNA isolated from normal donor feces mixed with 8E5 cells and the detection limit was as low as 2.5 copies/reaction (data not shown).

To test the sensitivity of amplifying HIV-1 RNA, 200 mg of normal donor feces were mixed with a series of different concentrations of HIV-1 positive plasma (with known HIV-1 RNA copies) and nucleic acid was isolated as before followed by RT nested PCR with HIV-1 env specific RT and PCR primers. As a human RNA input control in each sample, cDNA was synthesized by random hexamer followed by nested PCR amplification using human beta-actin mRNA specific primers. HIV-1 RNA was detected from normal feces mixed with HIV-1 positive plasma and the detection limit was as low as 40 copies/reaction (data not shown).

### Detection of HIV-1 DNA/RNA in feces

Four groups of study participants were recruited in 2008 (Table 1). **Group A**: 10 HIV-1 negative individuals; **Group B**: 11 HIV-1 infected individuals, who are drug naïve with detectable viral load in plasma ranging from 428 to 78,636 and CD4^+ ^T cell count ranging from 149 to 923. **Group C**: 13 HIV-1 infected individuals, who are on ART with undetectable viral load in plasma and CD4^+ ^T cell count ranging from 385 to 922. **Group D**: 5 HIV-1 infected individuals, who are on ART with detectable viral load in plasma ranging from 186 to 33,751 and CD4^+ ^T cell counts ranging from 152 to 540. Nucleic acids isolated from 200 mg feces were subjected to nested-PCR for amplification of HIV-1 env C2-V5 region. For every DNA sample, the human beta-globin gene was PCR amplified to ensure the comparable amount of human DNA contained in each PCR reaction. The beta-globin gene was detected in 17 out of 39 fecal specimens. Among these 17 beta-globin positive samples, one was from Group A, 8 from Group B, 3 from Group C and 5 from Group D. However, HIV-1 DNA was detected only in one fecal specimen from a patient (not on ART) with detectable viral load (XX495, viral load 35,471, Group B) (data not shown).

**Figure 1:**
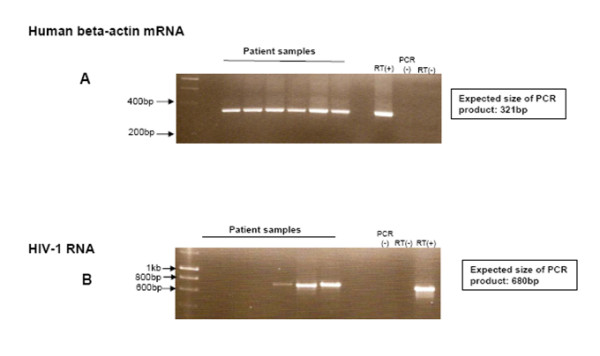
**Detection of HIV-1 RNA in feces collected from study participants**. A: Representative gel picture of RT nested PCR products of human beta-actin mRNA. B: Representative gel picture of RT nested PCR products of HIV-1 RNA.

Nucleic acids isolated from 200 mg of fecal specimen were subjected to RT nested-PCR. To serve as the human RNA input control for each sample, human beta-actin mRNA was amplified by RT nested PCR amplification. As shown in Figure 1A, relatively equal amounts of beta-actin mRNA were detected from all isolated fecal RNA, whereas HIV-1 RNA was detected in 3 out of 16 (19%) subjects with detectable viral load in blood (Figure 1A &1B).

### Confirmation of PCR specificity by using primers for another region (gag) of HIV-1 genome and sequencing the PCR products after cloning into a vector

To confirm the specificity of PCR amplification of env region in feces samples, the HIV-1 gag region was also amplified in these samples using gag specific primers. Two out of three HIV-1 env targeted-PCR positive and two out of two env negative fecal RNA samples maintained the identical outcome using gag specific primers. Furthermore, cloning and sequencing of the PCR product using env specific primers from the fecal samples demonstrated that the PCR products were HIV-1 subtype B env sequences (data not shown).

### Detection of human CD4 mRNA from the fecal samples

**Figure 2:**
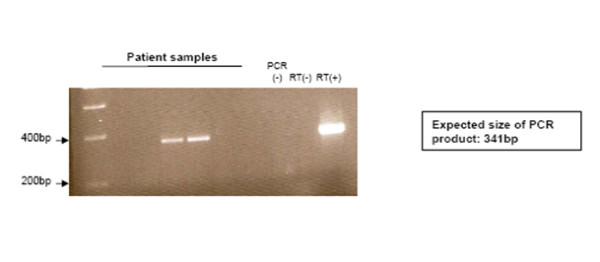
**Detection of CD4 mRNA in feces collected from study participants**. Representative gel picture of RT nested PCR products of CD4 mRNA from the MACS donor feces.

To monitor CD4 mRNA contained in the fecal samples, nucleic acids isolated from the fecal samples were subjected to RT nested PCR using CD4 mRNA specific primers. Human CD4 mRNA specific 110 bp PCR product was detected in 5 out of 16 (32%) subjects with detectable viral load in blood (Figure 2). Three of the detected 5 subjects were not on ART and 2 subjects were on ART. In contrast, no CD4 mRNA was detected in any HIV-1 uninfected donor's fecal specimens or infected donors with undetectable viral loads.

### Detection of fecal occult blood from the fecal samples

To detect the possible blood content in the fecal samples, an occult blood test was performed in all fecal specimens as described in Materials and Methods section. As shown in Table 3, fecal blood was detected in 7 out of 39 fecal specimens: 2 from Group A, 1 from Group B, 3 from Group C and 1 from Group D. The fecal occult blood test was negative for the fecal samples which were positive for HIV-1 RNA or DNA. One of the five CD4 mRNA positive fecal specimens was positive for occult blood and the remaining samples were negative.

**Table 3:** Summary of detection of HIV-1 RNA/DNA/human CD4 mRNA/fecal occult blood from feces and urines collected from MACS study participants in 2008

	**ID**	**Viral Loads (copies/ml)**	**CD4/mm**^3^	**URINE**		**FECES**			
				
				**HIV-1DNA**	**HIV-1 RNA**	**HIV-1 DNA**	**HIV-1 RNA**	**CD4** **mRNA**	**Fecal occult blood**
**Group A** **(N = 10)**	XX271	N/A	828	-	-	-	-	-	+
	
	XX744	N/A	1520	-	-	-	-	-	+

**Group B** **(N = 11)**	XX053	78636	406	-	-	-	-	-	+
	
	XX280	10003	923	-	-	-	-	+	-
	
	XX495	35471	238	-	-	+	-	-	-
	
	XX326	20149	392	-	-	-	+	-	-
	
	XX286	2974	320	-	-	-	-	+	-
	
	XX119	18231	901	-	+	-	+	+	-
	
	XX200	85820	149	+	-	-	-	-	-
	
	XX013	20441	457	+	-	-	-	-	-

**Group C** **(N = 13)**	XX245	<50	566	-	-	-	-	-	+
	
	XX690	<50	385	-	-	-	-	-	+
	
	XX265	<50	478	+	-	-	-	-	+
	
	XX523	<50	696	+	-	-	-	-	-
	
	XX327	<50	426	+	-	-	-	-	-

**Group D** **(N = 5)**	XX099	842	153	-	-	-	-	+	+
	
	XX274	16862	279	+	-	-	+	+	-
	
	XX371	33751	152	+	-	-	-	-	-

### Detection of HIV-1 DNA/RNA from the urine samples

The HIV *env *region was amplified from the DNA purified from the urine pellet in a nested-PCR reaction using the env primers described previously. For each DNA sample, human beta-globin gene was also PCR amplified to ensure the comparable amount of human DNA contained in each PCR reaction. All 34 urine pellet samples were positive for beta-globin amplification. HIV-1 DNA was detected in 7 urine pellet samples from HIV infected subjects, 4 from subjects with detectable viral load, and 3 from subjects with undetectable viral load (Table 3). Furthermore, RNA purified from the 34 urine supernatants was tested by RT nested-PCR to detect HIV *env *region. HIV-1 RNA was detected in one urine sample from a patient with detectable viral load.

### Sequence analysis of the HIV-1 envelope gene amplified from serum, feces and urine samples from an HIV-1 infected subject

**Figure 3:**
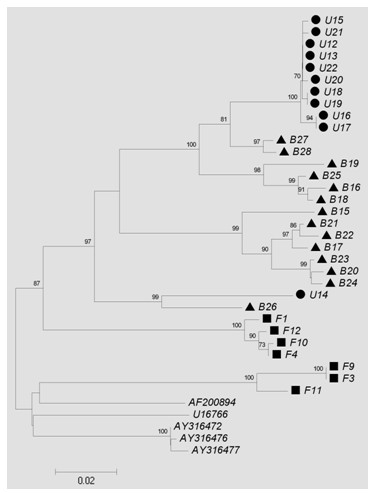
**Phylogenetic analysis of HIV-1 gp120 C_2_-V_5 _sequences from blood plasma, urine and feces**. Black triangles: sequences from blood; Black circles: sequences from urine; Black square: sequences from feces.

In one subject from Group B with viral load 18,231 copies/ml in blood, HIV-1 *env *was PCR detected in all three samples collected concurrently: serum, urine and feces. The PCR products corresponding to the C_2_-V_5 _region of HIV-1 *env *gp120 from all three compartments were cloned and sequenced to determine the viral diversity. A phylogenetic tree was constructed by the neighbor-joining method as implemented in Mega 4.0. The validity of the branching orders was estimated with 1000 replicates. Reference strains were obtained from the Los Alamos HIV database by using similar blast search. Phylogenetic analysis revealed that all the sequences belong to HIV-1 subtype B. Blood-, fecal- and urine- derived sequences formed a tightly clustered group of sequences respectively. However, the sequence analysis also showed a distributed pattern of viral variants among blood, feces and urine, indicating that distinct HIV-1 quasispecies existed in different part of tissues within the subject (Figure 3).

## Discussion

Many viral pathogens have been detected in feces, such as astrovirus, rotavirus, coronavirus, calcivirus, adenovirus and hepatitis C virus[20,21]. However, no comprehensive studies have been performed so far on the fecal samples from HIV-1 infected subjects. Furthermore, no studies have evaluated HIV-1 and CD4 mRNA level in feces from subjects at chronic stages of disease with or without ART.

GALT contains an abundant amount of CD4^+ ^T cells to maintain the mucosal immunity and is an important tissue for HIV replication in HIV infection. Ideally to monitor HIV-1 related GALT pathogenesis, a GI biopsy should be performed regularly to evaluate the dynamic changes of CD4^+ ^T cells and viral load in GALT. However, such studies are very difficult and expensive to implement. We hypothesized that during the massive HIV-1 replication and CD4^+ ^T cell depletion in GALT, HIV-1 and CD4^+ ^T cells shed into the intestinal lumen and the amount of HIV-1 and CD4^+ ^T cells in the feces would associate with the pathogenic changes in GALT.

Since the components of human feces are very complex and so far no sensitive methods are available for detection of viral and human RNA/DNA in human feces, we have initially tested assay sensitivity for nucleic acid isolation and detection of HIV-1 DNA/RNA in feces. Our results showed that HIV-1 DNA was detected from normal donor feces mixed with 8E5 cells with the detection limit of 2.5 copies of HIV-1 DNA/reaction. HIV-1 RNA was detected from normal feces mixed with HIV-1 positive plasma with detection limit of 40 copies of HIV-1 RNA/reaction. To evaluate usage of fecal specimens to monitor HIV-1 associated GALT pathogenesis, we collected the feces samples from HIV-1 uninfected and infected donors with or without ART.

In the 49 fecal samples collected in 2007 and 2008, HIV-1 DNA was detected in 1 subject from Group B (HIV-1 infected but not on ART) with detectable viral load of 35,471 copies/ml in plasma. HIV-1 RNA was detected in 4 subjects from Group B with viral load ranging between 14,690 and 55,396 copies/ml in plasma and in one subject from Group D (HIV-1 infected on ART) with detectable viral load of 16,862 copies/ml in plasma. Specificity of the HIV-1 detection was confirmed by amplifying HIV-1 *gag *region in these *env *positive samples. In the selected 5 feces samples, identical results were observed in 4 samples with the *gag *primers. One HIV-1 *env *positive feces sample was detected negative with *gag *primers. This might be due to the extremely low copy number of HIV-1 genomes contained in the sample, which could lead to limited PCR detection in the multiple PCR amplifications. Hoek at al[8] reported that no HIV-1 proviral DNA was detected, but HIV-1 RNA was detected in 67% of subjects' fecal samples by RT-nested PCR. Since the subjects involved in their study were in early stages of HIV-1 infection, when the rapid replication of HIV-1 and destruction of lymphoid tissues occurred in GALT, levels of HIV-1 shedding from GALT to intestinal lumen might be higher compared to the later chronic phase of infection.

The gastrointestinal tract is the major reservoir of viral infected cells and the site of rapid and profound loss of CD4 T cells, which could be the result from HIV direct killing of CD4-expressing primarily infected cells, HIV indirect killing of bystander cells through HIV proteins and/or by the proinflammatory state that is associated to ongoing viral replication[22]. One subset of CD4+ T cells in GALT is Th17 cells characterized by the production of IL-17, which are involved in epithelial regeneration and membrane barrier function. HIV/SIV-mediated Th17 depletion from GALT[23] impairs the gastrointestinal barrier and leads to translocation of intestinal microbes or microbial products, which then contribute to immune activation and disease progression[24,25]. We hypothesize that during CD4 T cell depletion, the depleted cells could shed into intestinal lumen through the impaired GI barrier and be discharged in feces. Because of the persistent active viral replication in GALT, CD4 positive cells are constantly lost from the GI tract throughout infection. Since cells and cellular proteins are degraded very quickly in the lumen of gastrointestinal tract, CD4 mRNA in feces has been monitored instead as a surrogate marker to evaluate CD4 T cells contained in the feces. However, it is possible that some of the detected CD4 mRNA were from macrophages and dendritic cells since these cells express CD4 as well.

All feces samples were screened for CD4 mRNA, a surrogate marker for CD4^+ ^T cells. No CD4 mRNA was detected in any feces samples from the HIV-1 negative donors or HIV-1 infected with undetectable plasma viral load, but CD4 mRNA was detected in 5 out of 16 fecal samples from the subjects with detectable plasma viral load, 3 from group B (HIV-1 infected not on ART) and 2 from group D (HIV-1 infected on ART with detectable plasma viral load). These results suggest that CD4^+ ^T cells were shed from GALT to intestinal lumen in the infected subjects with detectable plasma viral load.

Detection of blood in feces has long been regarded as an indicator of subject's state of health[26]. HIV-1 RNA/DNA and CD4 mRNA detected in the fecal specimens of HIV-1 infected subjects could be the result either from internal bleeding in the gastrointestinal tract or from shedding of HIV-1 infected cells and free virus from GALT. To dissect these two possibilities, fecal occult blood test was performed in all samples. As listed in Table 3, a total of 7 fecal samples were positive in the test: 2 from Group A, 1 from Group B, 3 from Group C and 1 from Group D. All fecal samples positive for HIV-1 RNA/DNA were negative in the occult blood test. Thus, HIV-1 RNA/DNA detected in the fecal samples could be the result of the shedding of HIV-1 infected cells and/or free virus from GALT into the intestinal lumen.

HIV-1 has been detected in a variety of body fluids and secretions including blood, semen, vaginal fluids and breast milk. Li et al. [17] in 1992 reported the presence of HIV-1 DNA proviral sequences in fresh urine pellets from HIV-1 seropositive individuals. However, the presence of HIV-1 in urine from chronic infected subjects with or without ART has not been addressed. Our data show that HIV-1 DNA and RNA were detected in both the urine pellet and the supernatant from the HIV-1 infected subjects (Table 3). HIV-1 DNA was detected in 7 urine pellet samples from the 29 HIV-1 infected subjects, 4 of the 7 samples from subjects (2 subjects were not on ART and 2 subjects on ART) with detectable plasma viral load, and 3 from subjects (who were on ART) and with undetectable viral load. This result indicates that the detection of HIV-1 in urine is not necessarily associated with plasma viral load.

Due to the tissue-specific anatomical structures and local immunological components, virus replication in different tissues in infected individual could result in the diversity of HIV populations. It has been reported that HIV compartmentalization was present between blood and feces/gastrointestinal tissues[8-10,27], between blood and urogenital/genital tract [28-30], and SIV compartmentalization between blood and brain/cerebrospinal fluid[31,32]. In this study, the diversity of HIV-1 populations in three compartments (blood, GI tract and urine system) was evaluated. The sequencing analysis of HIV-1 *env *C_2_-V_5 _region from concurrently collected fecal, urine and blood specimens revealed that HIV-1 compartmentalization was present in the three compartments of this subject.

In summary, this cross-sectional study indicates that the sensitive PCR based method is suitable for HIV-1 DNA/RNA and CD4 mRNA detection in feces and urine samples from HIV-1 infected individuals. In addition, HIV-1 compartmentalization was revealed in gut, renal system and blood in one infected subject. The presence of HIV-1 RNA/DNA in fecal and urine samples from HIV-1 infected subjects is lesser in the chronic stage of infection compared to the acute stage of infection. Our results suggest that feces could be an informative specimen in clinic for detecting HIV and CD4 mRNA. Since a small sample size was used in the current study, future longitudinal studies are needed to show whether there is a correlation of detection of HIV-1 RNA/DNA and CD4 mRNA in feces with disease progression.

## Competing interests

The authors declare that they have no competing interests.

## Authors' contributions

AC carried out all the feces DNA/RNA isolation and HIV-1 detection work and drafted the manuscript. LC, MD and CS performed urine DNA/RNA isolation and HIV-1 detection, the molecular genetic studies and help to draft the manuscript. WB participated in the design of the study and samples collection. YC, PG, and CRR participated in its design and coordination and helped to draft the manuscript. All authors read and approved the final manuscript.

## Genebank Accession Numbers

The genebank accession numbers of the nuclotide sequences reported in this paper are GQ260025-GQ260055.
